# Assessing the Impact of PRESERFLO MicroShunt on Intraocular Pressure in Porcine Eyes Ex Vivo Using Infusion Pump System

**DOI:** 10.3390/bioengineering11070669

**Published:** 2024-06-29

**Authors:** Andi Masdipa, Sachiko Kaidzu, Masaki Tanito

**Affiliations:** Department of Ophthalmology, Shimane University Faculty of Medicine, Izumo 693-8501, Japan; m209402@med.shimane-u.ac.jp (A.M.); kecha@med.shimane-u.ac.jp (S.K.)

**Keywords:** glaucoma, PRESERFLO MicroShunt, ex vivo studies, intraocular pressure (IOP), porcine eyes, MIGS

## Abstract

To evaluate the effectiveness of the PRESERFLO MicroShunt (PFM) in reducing intraocular pressure (IOP) ex vivo in porcine eyes using an infusion pump system and to simulate various IOP conditions, In this study, porcine eyes received increasing flows between 2 and 20 μL/min. IOP measurements were taken under conditions with and without the PFM [PFM (+) and PFM (−), respectively]. In the PFM (−) group, IOP increased from 7.4 mmHg to 46.3 mmHg as the flow rate increased from 2 μL/min to 20 μL/min. The rate of IOP reduction (%ΔIOP) rose with increasing flow rates, although the absolute IOP values achieved with the PFM insertion also increased. The correlation between IOPs in the PFM (−) conditions and the %ΔIOP was modeled as %ΔIOP = 22.4 Ln [PFM(−) IOP] − 41.7. According to this equation, IOP reduction by PFM insertion is 0% at IOPs of 6.4 mmHg or lower. IOP reductions of 10%, 20%, 30%, and 40% were observed when the pre-insertion IOPs were 10.1, 15.7, 24.6, and 38.4 mmHg, respectively. Achievable post-insertion IOP levels of ≤21 mmHg, ≤18 mmHg, ≤15 mmHg, and ≤12 mmHg corresponded to the initial IOPs of 33 mmHg, 26 mmHg, 20 mmHg, and 14.8 mmHg, respectively. In conclusion, the PFM effectively reduced IOP within a specific range of IOP values in an ex vivo experimental system. In clinical situations, the PFM is unlikely to be effective at low IOP levels. At higher levels, the PFM reduces IOP, but it may be insufficient to achieve the target IOP.

## 1. Introduction

The PRESERFLO MicroShunt (PFM) represents a significant advancement in the surgical management of glaucoma, offering a novel approach to reducing intraocular pressure (IOP) by facilitating the drainage of aqueous humor (AH) into the subconjunctival space [1,2]. Its micro-scale design enables improved intraocular fluid management without necessitating complex surgical procedures, potentially revolutionizing glaucoma treatment strategies [3,4].

Previous research has highlighted the PFM’s effectiveness and identified potential complications associated with its use, with several studies documenting these outcomes in detail [5,6]. Moreover, in vivo research often employs rabbit eyes as a model to further understand the device’s performance and implications [5]. Building on our preceding work, which examined the pressure characteristics of the PFM on scleral segments of porcine eyes [6], our current investigation seeks to expand this knowledge base. The Hagen–Poiseuille law was employed to calculate the resistance offered by the PFM, an essential factor since the PFM dictates the pressure resistance encountered as fluid traverses through a capillary. This study focuses on determining the optimal resistance range for the PFM, addressing a gap in experimental data regarding its application.

The primary objective of our study is to evaluate the PFM’s efficacy in reducing IOP when implanted into porcine eyes ex vivo, utilizing an infusion pump system for precise measurement. Although porcine eyes exhibit notable differences from human eyes, they share several anatomical and physiological similarities, such as size, making them a valuable model for ophthalmological research [7,8]. These similarities enable porcine eyes to serve as an effective proxy for human eyes in ex vivo studies, providing insights that are likely applicable to human conditions. Our research question investigates whether the PFM can achieve the desired target IOP across varying levels of initial IOP, aiming to identify a specific IOP threshold where the device is most effective.

## 2. Materials and Methods

### 2.1. Materials

This study utilized six PRESERFLO MicroShunts (PFM) provided by Santen Pharmaceutical Co., Osaka, Japan. Seven whole porcine eyes were sourced from local slaughterhouses on the day of the experiment and transported to our laboratory within eight hours post-enucleation to prevent tissue degradation. Notably, the use of these eyes falls outside the scope of animal welfare regulations pertaining to live animal experiments [6]. Additional materials included disposable 27G ^3^/_4_ inch needles and 1 mL syringes from Terumo Corporation, Tokyo, Japan; infusion tubes from JMS Co., Ltd., Tokyo, Japan; and a syringe pump (SP101i) from Kd Scientific, Holliston, MA, USA. Pressure measurement equipment comprised a BLPR2 pressure transducer, a 4-channel amplifier (SYS TBM4M), an analog-to-digital converter (LAB-TRAX-4/16), and LabScribe2 software for pressure curve analysis, all from World Precision Instruments, Sarasota, FL, USA. A specialized two-step gap knife from MANI, Inc., Tochigi, Japan, was employed for scleral incisions.

### 2.2. Experimental Setup

**Figure 1:**
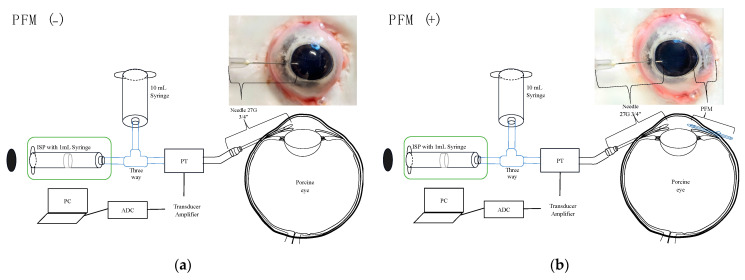
Experimental settings of PFM (−) (**a**) and PFM (+) (**b**) conditions. ISP, infusion syringe pump; PT, pressure transducer; PC, personal computer; ADC, analog-to-digital converter.

**Figure 2:**
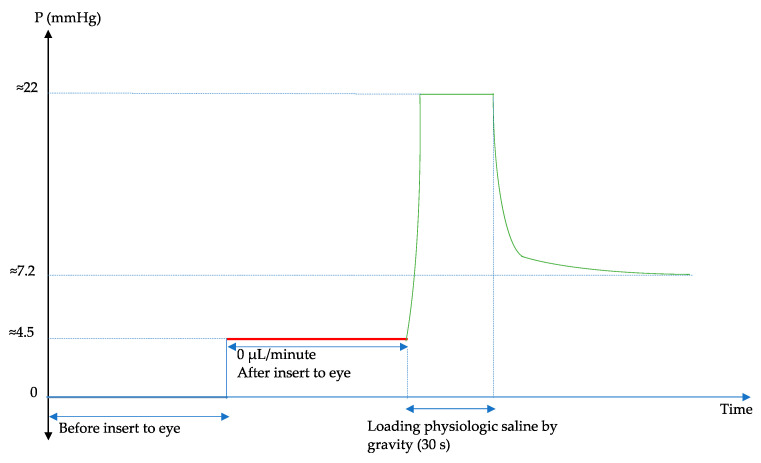
Illustration of the preparation and loading of physiologic saline into a porcine eye.

The setup for pressure measurements replicated that of our prior studies [6,9]. The infusion syringe pump was prepared with a 1 mL syringe, connected to the pressure transducer via infusion tubing, and linked to a 27G needle. This assembly was then connected to a transducer amplifier, an analog-to-digital converter, and finally to a computer for IOP recording (Figure 1a). Initial calibration involved filling the needle tip with physiological saline and adjusting the pump until the saline’s surface pressure read zero (Figure 2).

Next, the porcine eyes were positioned on a cork base and carefully secured with needles to avoid exerting pressure that could influence IOP. Following the release of the conjunctiva from the sclera using a surgical set, a 27G ^3^/_4_ inch needle was inserted 3 mm from the limbus (Figure 1a). Once the needle entered the anterior chamber, the pressure increased by approximately 4.5 mmHg, which also reflected the IOP of the eye (Figure 2). Due to the low IOP, physiological saline was introduced into the anterior chamber by gravity from a 10 mL syringe by adjusting the three-way tap (Figure 1), resulting in an IOP reading of about 22 mmHg (Figure 2). After 30 s, the introduction of physiological saline was halted, and the three-way tap was returned to its initial setting. It was observed that the pressure rapidly decreased then slowed until it stabilized, indicating an IOP value of approximately 7.2 mmHg (Figure 2). This pressure decrease became steady after the loading process, recorded as the initial pressure at a flow rate of 0 μL/min. Subsequently, the experiment proceeded by setting the flow rate to 2 μL/min for 5 min, during which IOP measurements were taken. This procedure was repeated at 5 min intervals with an increase of 2 μL/min in the flow rate until a maximum rate of 20 μL/min was reached.

After completing measurements without a PFM (PFM (−) condition), a PFM was inserted into the sclera on the opposite side of the initial needle insertion point (PFM (+) condition) (Figure 1b). The integrity of the PFM was verified by observing saline droplets at the distal end following a flush.

### 2.3. Data Analysis

The data analysis was performed using JMP Pro 17 statistical software (JMP Statistical Discovery, Cary, NC, USA). All data are presented as the mean and standard deviation (SD). Comparisons between the PFM (−) and PFM (+) conditions were tested using the paired *t*-test. A possible correlation between IOPs in the PFM (−) condition and IOP reduction achieved in the PFM (+) condition was assessed by log curve fitting.

## 3. Results

**Figure 3:**
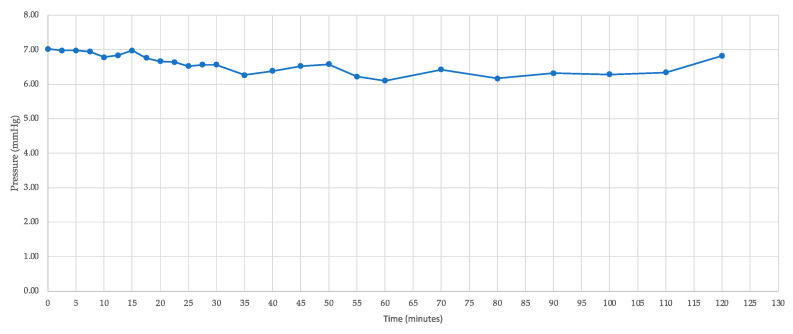
Graph of IOP measurement in porcine eyes with a flow of 2 μL/min for 2 h.

The IOP of seven porcine eyes was accurately recorded following gravitational loading with physiological saline. One eye was subjected to a continuous flow of 2 μL/min for 2 h, during which IOP was recorded at intervals ranging from 2.5 to 10 min (Figure 3, Table 1). This process yielded IOP values between 6.1 mmHg and 6.9 mmHg, with an average IOP of 6.5 ± 0.3 mmHg. The narrow range of 0.8 mmHg and the low standard deviation demonstrate the consistency and stability of the IOP measurements in this experimental context.

**Figure 4:**
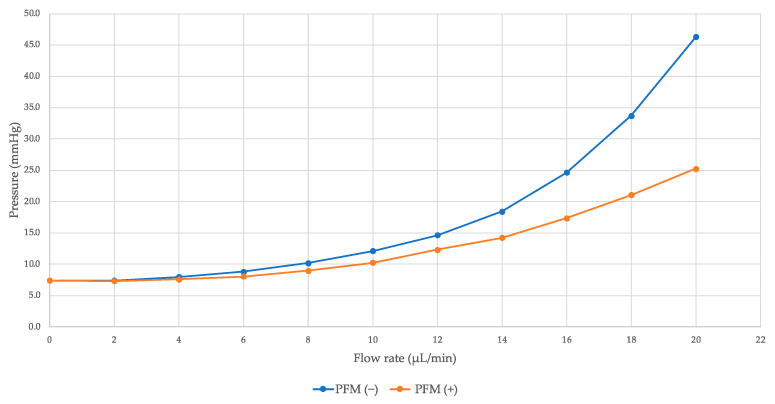
Measured IOPs with different flow rates in PFM (−) and PFM (+) conditions.

For the other six porcine eyes, IOP was measured at 5 min intervals with a gradual increase in flow rate from 2 μL/min to 20 μL/min. This method allowed for the generation of a range of IOP values, facilitating a comparison of measured IOPs between conditions without the PFM (PFM (−)) and with PFM (PFM (+)). In the PFM (−) group, IOP escalated from 7.4 mmHg to 46.3 mmHg as the flow rate increased from 2 μL/min to 20 μL/min (Table 2). As the flow rate rose, the percentage of IOP reduction (%ΔIOP) also increased, although the IOP levels achieved post-PFM insertion were higher (Figure 4).

**Figure 5:**
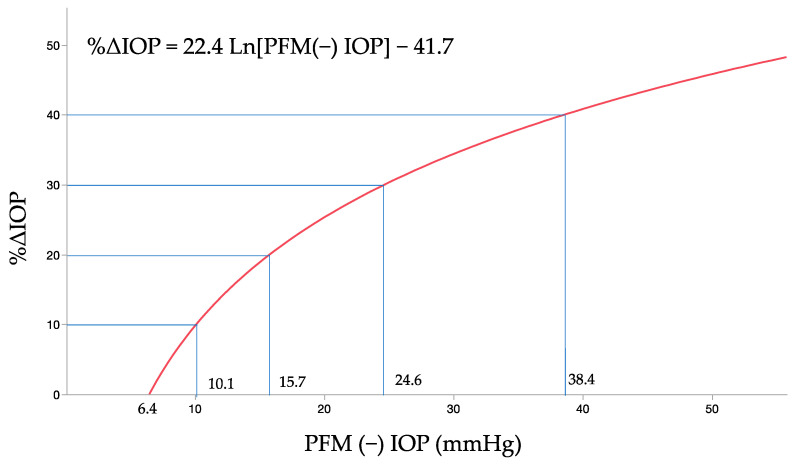
Fitted curve for association between PFM (−) pressure and %IOP decrease (%ΔIOP) observed in PFM (+) condition. Theoretically, the reduction in IOP attributable to the PFM insertion is quantified as 0%, 10%, 20%, 30%, and 40% when the pre-insertion (PFM (−)) IOP values are at 6.4, 10.1, 15.7, 24.6, and 38.4 mmHg, respectively. Consequently, post-PFM insertion, the achievable pressure levels of ≤21 mmHg, ≤18 mmHg, ≤15 mmHg, and ≤12 mmHg correspond to pre-insertion pressures of 33, 26, 20, and 14.8 mmHg, respectively.

The curve fit analysis of the collected IOP data revealed a correlation between the IOPs in the PFM (−) condition and the %ΔIOP due to the PFM insertion, described by Equation (1) (Figure 5):

%ΔIOP = 22.4 Ln [PFM(−) IOP] − 41.7(1)

According to this equation, the IOP reduction attributable to the PFM insertion drops to 0% for IOP values of 6.4 mmHg or lower in the PFM (−) condition. IOP reductions of 10%, 20%, 30%, and 40% are observed when the pre-insertion IOP levels are at 10.1, 15.7, 24.6, and 38.4 mmHg, respectively. Furthermore, post-insertion IOP levels of ≤21 mmHg, ≤18 mmHg, ≤15 mmHg, and ≤12 mmHg can be achieved when the initial IOP levels are at 33 mmHg, 26 mmHg, 20 mmHg, and 14.8 mmHg, respectively.

## 4. Discussion

In this ex vivo study, the PFM was inserted into porcine eyes to serve as a fluid drain from the anterior chamber, demonstrating an increasing percentage decrease in IOP as the measured IOP value rose. This variation in IOP decrease was dependent on the flow rate of physiologic saline directed into the eye.

In vivo, AH production by the ciliary body and its drainage through both conventional and unconventional pathways maintain eye pressure. The average AH production rate in humans is around 2–3 µL per minute [10,11]. In this study, physiologic saline was substituted for AH, regulated via syringe pump infusion, following Goldmann’s equation, Equation (2), for AH dynamics [12,13]:

(2) $$F=\left(P_{i}-P_{e}\right)\times C+U$$

where *F* denotes the rate of AH formation; *U* represents unconventional AH outflow; $P_{i}$ is the IOP; *C* indicates the conventional outflow facility; and *P_e_* is episcleral venous pressure.

For the purpose of this study, we used enucleated porcine eyes, assuming *P_e_* to be absent. Furthermore, assuming that C remains constant across all conditions and that U changes with PFM insertion, F and U become the variables influencing the measured IOP in this experiment. Given that the normal range of IOP in human eyes is approximately 10–21 mmHg [14,15], providing graded F values was a method to achieve a spectrum of IOP values to assess their correlation with PFM insertion. Consequently, Equation (2) can be adapted into Equation (3) for IOP [12]:

(3) $$IOP=\frac{\left(F-U\right)}{C}+P_{e\;}$$

In the equation above, a higher F value correlates with a higher measured IOP, and changes in U occur following the insertion of the PFM. Normally, AH production is balanced by its outflow, maintaining a stable IOP [12,13]. When F and U are equivalent, the IOP remains unchanged. This scenario was observed in the PFM (−) condition after administering a 2 µL/min flow. In the initial 5 min of this flow, the IOP remained stable at 7.4 mmHg, as evidenced by the data in Table 1, where a 2 µL/min infusion was maintained for 2 h, with IOP measurements recorded every 2.5 min, demonstrating consistent stability as shown in Figure 4. This finding indicates that a 2 µL/min physiologic saline flow into the anterior chamber does not alter IOP. However, in the PFM (+) condition, after a 5 min flow at 2 µL/min, the IOP did not decrease from 7.4 mmHg to 7.3 mmHg. According to the derived equation, IOP reduction by the PFM is nonexistent at 6.4 mmHg or lower in the PFM (−) condition. This outcome suggests that the PFM, acting as a drainage mechanism post-insertion, increases outflow from the anterior chamber beyond the inflow of physiologic saline from the pump, indicating that at low IOP levels, the PFM does not effectively maintain IOP.

In this study, the variation in measured IOP values resulted from administering physiologic saline at varying flow rates. Following the insertion of the PFM, a reduction in IOP was observed in the PFM (+) condition compared to the pre-insertion values in the PFM (−) condition. Clinical benchmarks for successful PFM insertion have been defined as achieving an IOP of less than specific IOP levels (e.g., <21 mmHg, <18 mmHg, <15 mmHg, and 12 mmHg) and a percentage IOP reduction exceeding 20% [16,17]. A reduction exceeding 20% was noted when the IOP changed from 18.4 mmHg in the PFM (−) condition to 14.3 mmHg in the PFM (+) condition, equating to a 22.6% reduction at a flow rate of 14 μL/min, as detailed in Table 2. This finding is in line with a meta-analysis that reported an IOP reduction from 21.7 ± 8.4 mmHg to 15.9 ± 8.5 mmHg using the PFM, marking a 26.7% decrease [18], mirroring our observations. The data elucidated by the equation underscore that the levels of IOP prior to PFM insertion have a direct impact on the percentage of IOP reduction and the post-insertion IOP levels. Concisely, an IOP reduction greater than 20% begins at initial IOP levels of approximately 15 mmHg and above, while achieving post-insertion IOP levels below 21 mmHg necessitates initial IOP levels around 30 mmHg or lower.

Previous research has demonstrated a similar correlation following goniotomy procedures [19,20]. Conversely, such a direct relationship is not consistently observed after trabeculectomy, where an IOP reduction can be achieved regardless of whether the preoperative IOP was high or low [21]. Although the PFM is classified as a type of filtration surgery, its relationship between preoperative and postoperative IOP levels seems akin to that observed with goniotomy, suggesting a potentially predictive model for postoperative outcomes based on preoperative IOP levels.

Recognizing the limitations of this study is crucial, particularly the use of the porcine eye model, which does not incorporate specific outflow obstruction characteristic of glaucomatous eyes. Consequently, the measured IOP values might not fully translate to the outcomes expected in human eyes following PFM insertion, especially considering that the distal end of the PFM is not enveloped by the conjunctiva in the model used. Additionally, the study did not account for tissue reactions such as scarring, inflammation of the AH, fibrin formation, and bleeding, which could influence the efficacy and safety profile of the PFM in a clinical setting. Moreover, the methodology employed to induce variations in IOP was designed to simulate a range of pressures rather than to replicate the specific pathophysiological conditions of glaucoma, where a decrease in AH outflow typically precipitates elevated IOP. Despite these constraints, the experimental findings delineate a specific IOP range wherein the PFM demonstrates optimal functionality. However, it is important to consider that external factors not identified or controlled for in the porcine model may affect the validity and generalizability of the results to human clinical practice.

## 5. Conclusions

Although the enucleated porcine eyes utilized in this study significantly differ from living human eyes, the observed variations in IOP following the administration of physiologic saline flow, and the subsequent decrease in IOP post-PFM insertion, affirm that the PFM operates effectively within a particular range of IOP values. Specifically, at low IOP levels, the insertion of the PFM does not influence IOP, whereas at elevated IOP levels, PFM insertion results in a reduction in IOP values, but potentially surpasses the desired IOP target. Looking ahead, it is recommended that future research endeavors investigate the potential effects of varying the positions and angles at which the PFM is utilized to further understand and optimize its application and effectiveness.

## Figures and Tables

**Table 1:** IOP measurement in a porcine eye with a flow of 2 μL/minute for 2 h.

Time	IOP (mmHg)
0	6.9
10	6.8
20	6.7
30	6.6
40	6.5
50	6.7
60	6.1
70	6.4
80	6.2
90	6.3
100	6.3
110	6.3
120	6.8
Mean ± SD	6.5 ± 0.3

IOP, intraocular pressure; SD, standard deviation.

**Table 2:** IOP measurements in porcine eyes (*N* = 6) under PFM (−) and PFM (+) conditions.

Flow (µL/Minute)	IOP (mmHg)		%ΔIOP	*p* Value
	PFM (−)	PFM (+)		
0	7.4 ± 0.4	7.4 ± 0.4	0.0%	>0.99
2	7.4 ± 0.4	7.3 ± 0.5	1.0%	0.46
4	8.0 ± 0.7	7.6 ± 0.7	4.4%	0.11
6	8.8 ± 0.9	8.1 ± 0.8	8.8%	0.0011
8	10.2 ± 1.4	9.0 ± 1.0	12.0%	0.0082
10	12.1 ± 1.9	10.2 ± 1.5	15.6%	0.0099
12	14.6 ± 2.2	12.3 ± 2.4	15.8%	0.0041
14	18.4 ± 3.4	14.3 ± 3.6	22.6%	0.0033
16	24.7 ± 6.1	17.4 ± 5.1	29.5%	0.0014
18	33.7 ± 9.5	21.1 ± 7.0	37.6%	0.0009
20	46.3 ± 14.7	25.3 ± 8.9	45.4%	0.0009

%ΔIOP, percent IOP reduction. Data are expressed in mean ± standard deviation. *p* values are calculated by paired *t*-test.

## Data Availability

Data are fully available upon reasonable request to the corresponding author.

## References

[1] Armstrong J.J., De Francesco T., Ma J., Schlenker M.B., Ahmed I.I.K. (2023). Ab Externo SIBS Microshunt with Mitomycin C for Open-Angle Glaucoma: Three-Year Results as a Primary Surgical Intervention. Ophthalmol. Glaucoma.

[2] Schlenker M.B., Armstrong J.J., De Francesco T., Ahmed I.I.K. (2023). All Consecutive Ab Externo SIBS Microshunt Implantations with Mitomycin C: One-Year Outcomes and Risk Factors for Failure. Am. J. Ophthalmol..

[3] Pinchuk L., Riss I., Batlle J.F., Kato Y.P., Martin J.B., Arrieta E., Palmberg P., Parrish R.K., Weber B.A., Kwon Y. (2016). The use of poly(styrene- block -isobutylene- block -styrene) as a microshunt to treat glaucoma. Regen. Biomater..

[4] Sadruddin O., Pinchuk L., Angeles R., Palmberg P. (2019). Ab externo implantation of the MicroShunt, a poly (styrene-block-isobutylene-block-styrene) surgical device for the treatment of primary open-angle glaucoma: A review. Eye Vis..

[5] Fujimoto T., Nakashima K.I., Watanabe-Kitamura F., Watanabe T., Nakamura K., Maki K., Shimazaki A., Kato M., Tanihara H., Inoue T. (2021). Intraocular Pressure-Lowering Effects of Trabeculectomy Versus MicroShunt Insertion in Rabbit Eyes. Transl. Vis. Sci. Technol..

[6] Masdipa A., Kaidzu S., Tanito M. (2023). Exploring the Pressure Characteristics of the PRESERFLO MicroShunt in In Vitro Studies and Effects of Sclera on Device Performance. J. Clin. Med..

[7] Middleton S. (2010). Porcine ophthalmology. Vet. Clin. North. Am. Food Anim. Pract..

[8] McMenamin P.G., Steptoe R.J. (1991). Normal anatomy of the aqueous humour outflow system in the domestic pig eye. J. Anat..

[9] Masdipa A., Kaidzu S., Tanito M. (2023). Flow Pressure Characteristics of the Ahmed Glaucoma Valve and Possible Effect of Entrapped Air in the Tube. Transl. Vis. Sci. Technol..

[10] McLaren J.W. (2009). Measurement of aqueous humor flow. Exp. Eye Res..

[11] Sunderland D.K., Sapra A. (2024). Physiology, Aqueous Humor Circulation. StatPearls.

[12] Brubaker R.F. (2004). Goldmann’s equation and clinical measures of aqueous dynamics. Exp. Eye Res..

[13] Zhao M., Hejkal J.J., Camras C.B., Toris C.B. (2010). Aqueous humor dynamics during the day and night in juvenile and adult rabbits. Investig. Ophthalmol. Vis. Sci..

[14] Weinreb R.N., Khaw P.T. (2004). Primary open-angle glaucoma. Lancet.

[15] Chen M., Zhang L., Xu J., Chen X., Gu Y., Ren Y., Wang K. (2019). Comparability of three intraocular pressure measurement: iCare pro rebound, non-contact and Goldmann applanation tonometry in different IOP group. BMC Ophthalmol..

[16] Crawley L., Zamir S.M., Cordeiro M.F., Guo L. (2012). Clinical options for the reduction of elevated intraocular pressure. Ophthalmol. Eye Dis..

[17] Majoulet A., Scemla B., Hamard P., Brasnu E., Hage A., Baudouin C., Labbé A. (2022). Safety and Efficacy of the Preserflo^®^ Microshunt in Refractory Glaucoma: A One-Year Study. J. Clin. Med..

[18] Pawiroredjo S.S.M., Bramer W.M., Pawiroredjo N.D., Pals J., Poelman H.J., de Vries V.A., Wolfs R.C.W., Ramdas W.D. (2022). Efficacy of the PRESERFLO MicroShunt and a Meta-Analysis of the Literature. J. Clin. Med..

[19] Tanito M., Sugihara K., Tsutsui A., Hara K., Manabe K., Matsuoka Y. (2021). Effects of Preoperative Intraocular Pressure Level on Surgical Results of Microhook Ab Interno Trabeculotomy. J. Clin. Med..

[20] Sakamoto T., Nisiwaki H. (2023). Factors associated with 1-year outcomes and transient intraocular pressure elevation in minimally invasive glaucoma surgery using Kahook Dual Blades. Sci. Rep..

[21] Ishida A., Miki T., Naito T., Ichioka S., Takayanagi Y., Tanito M. (2023). Surgical Results of Trabeculectomy among Groups Stratified by Prostaglandin-Associated Periorbitopathy Severity. Ophthalmology.

